# A New Fuzzy-Based Classification Method for Use in Smart/Precision Medicine

**DOI:** 10.3390/bioengineering10070838

**Published:** 2023-07-15

**Authors:** Elena Zaitseva, Vitaly Levashenko, Jan Rabcan, Miroslav Kvassay

**Affiliations:** Department of Informatics, Faculty of Management Science and Informatics, University of Zilina, 01026 Zilina, Slovakia; jan.rabcan@fri.uniza.sk (J.R.); miroslav.kvassay@fri.uniza.sk (M.K.)

**Keywords:** fuzzy classification, fuzzy decision tree, data types, smart medicine, Medicine 4.0

## Abstract

The development of information technology has had a significant impact on various areas of human activity, including medicine. It has led to the emergence of the phenomenon of Industry 4.0, which, in turn, led to the development of the concept of Medicine 4.0. Medicine 4.0, or smart medicine, can be considered as a structural association of such areas as AI-based medicine, telemedicine, and precision medicine. Each of these areas has its own characteristic data, along with the specifics of their processing and analysis. Nevertheless, at present, all these types of data must be processed simultaneously, in order to provide the most complete picture of the health of each individual patient. In this paper, after a brief analysis of the topic of medical data, a new classification method is proposed that allows the processing of the maximum number of data types. The specificity of this method is its use of a fuzzy classifier. The effectiveness of this method is confirmed by an analysis of the results from the classification of various types of data for medical applications and health problems. In this paper, as an illustration of the proposed method, a fuzzy decision tree has been used as the fuzzy classifier. The accuracy of the classification in terms of the proposed method, based on a fuzzy classifier, gives the best performance in comparison with crisp classifiers.

## 1. Introduction

**Figure 1:**
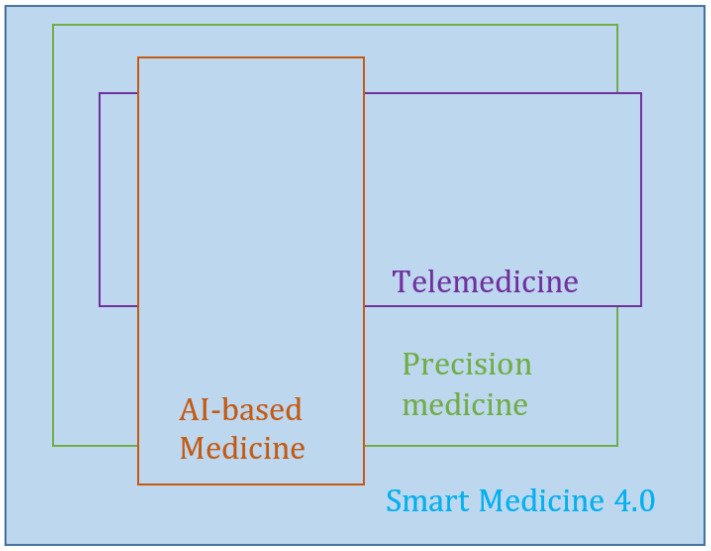
The structure of Medicine 4.0.

There is a global consensus about the relevance of information technology (IT) for healthcare, within which it now plays an integral part [1,2,3]. Therefore, the creation of concepts or approaches that have a significant impact on IT also has a strong impact on medical applications. The first IT-based applications in medicine were associated with the use of decision-support systems and image processing, a document management system for medical records, and the development of evidence-based medicine [4]. The concepts of big data, the Internet of Things (IoT), cloud computing, artificial intelligence (AI), and other inventions have transformed modern technologies under Industry 4.0. The development of IT in recent times has resulted in such phenomena as “precision medicine”, “smart medicine” or “Medicine 4.0” [3,5,6,7]. According to previous studies [3,5], an integral part of Medicine 4.0 or smart medicine is the use of AI-based applications in medicine (AI-based medicine), telemedicine, and precision medicine (Figure 1). AI-based medicine includes the most frequently used applications in healthcare, which allow the analysis of medical images and clinical and laboratory data. Artificial intelligence, including machine learning (ML) and big data processing, supports diagnosis and treatment in the healthcare environment [7,8,9]. Telemedicine using IoT will make seeing a patient or being seen by a clinician easier. Based on these technologies, such applications in the context of healthcare as electronic health records (EHR) [10], wearable devices (pulse oximeters or glucose monitoring) [5], and others have been developed. Precision medicine guides the course of treatment by using more comprehensive molecular diagnoses, such as genotyping or RNA expression.

The structure of Medicine 4.0, proposed by the authors of [3,5] (Figure 1), is created by data types, which are diverse in their nature and are obtained from various sources. These differences necessitate different types of data analysis, which, in turn, leads to different methods for processing. For example, the analysis of medical images requires methods to be in place for image processing and analysis; these methods cannot be those that are applied to expert data analysis and evaluation in the form of the physicians’ notes drawn from EHR. The sharing of various IT methods, which are traditionally developed independently, is typical for medical applications. This problem can be considered partly from the point of view of the concept of big data. In their studies [2], the authors have shown that the principal impact on precision medicine has been the paradigm of big data. The role of big data has increased in healthcare and needs new approaches and methods for medical data analysis, in an environment where individual patient data, including their genes, environment, and lifestyle, are taken into account to identify the most suitable treatment. The methods for such data processing and analysis are mostly drawn from AI and ML.

**Figure 2:**
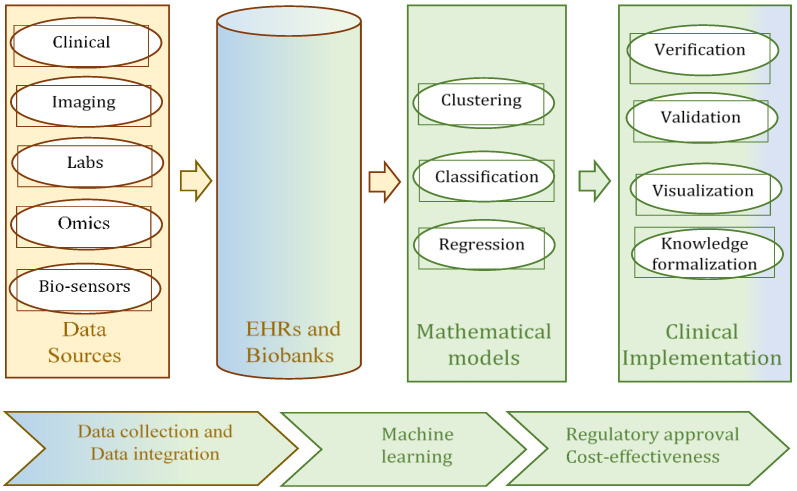
Typical data types and ML methods for their processing and analysis.

AI in the context of health is used for a wide range of problems, from retrospective to prospective analysis. The methods and approaches of AI depend on many factors, such as the goal of the analysis (for example, data description, diagnostics, prediction, or prescription), data types (numeric, discrete, time factors, etc.), uncertainty (crisp or uncertain data, whether completely or incompletely specified) and other. The taxonomy of these problems, the methods, and the specifics of the data are illustrated in Figure 2 [11]. Four groups of applications, sorted according to this taxonomy, can be identified as relevant to healthcare, and precision medicine in particular, at the present time. The first of these groups can be identified as the collection of information from repositories, EHR, or biobanks [10]. The second and third groups can be interpreted as data analysis problems; the principal methods for their decision-making processes are clustering, classification, and regression (Figure 2), i.e., methods of ML. The initial data for these methods can be collected as data from biobanks and EHRs [12]. These methods enable the extraction of clinically meaningful insights from the data. Most medical applications currently deliver services within these three groups. In other words, these are effective methods and services for data collection, representation, preliminary analysis, and processing, in order to obtain clinically meaningful information for physicians. The fourth group of applications in healthcare is mostly present in the educational domain. The different simulators typically act as learning tools for use in medical universities [13,14].

As has been shown in several recent reviews [7,12,15,16], methods of classification, clustering, and regression are commonly used for medical applications (Figure 2). In some studies [17,18], association rules are considered to be one more alternative as a mathematical model for data analysis. They allow the identification of patterns, correlations, and causal patterns in the investigated samples. Among these methods, classification is the most frequently and successfully used technique. The greatest advances have been made in medical applications in which classification has been implemented based on neural networks or deep learning [19,20,21], decision trees or hierarchical analysis [22], and linear regression [23]. The most frequently used classification technologies in precision medicine are decision tree-based and neural network-based. The efficiency and advantages of the decision tree-based classifier lie in these factors: the classification result has good visualization and easy interpretation; a classifier can be inducted based on data with a small number of samples and/or a small number of attributes; there is no requirement to normalize the initial data. The undoubted advantages of neural networks are very good classification accuracy and the ability to process data with a very large number of samples. The only significant drawback of neural networks (deep learning) from the point of view of precision medicine is its representation in the form of a “black box”, which does not allow researchers to retain at least some interpretability of the classification result. In some cases, to eliminate this shortcoming of neural networks, several classifiers are used simultaneously. There are studies in which an ensemble of classifiers is used. For example, in one study [24], random forests, an artificial neural network (ANN), and a support vector machine (SVM) were used for COVID-19 outbreak prediction. Therefore, despite the obvious advantages offered by neural networks, quite often, developers turn to such classifiers as decision trees [22,25,26], random forests [27,28], and fuzzy decision trees [29,30]. These classifiers offer good interpretability of the classification results, which is very important in precision medicine applications.

However, on the one hand, there are neural networks that offer good efficiency and applicability to big data, while on the other hand, there are decision tree-based classifiers that offer good interpretability of the classification result. The development of precision medicine applications should keep in mind the specifics of initial data for classifier induction. The data in precision medicine are of different types (heterogeneous). These data can be incomplete and uncertain, which is considered to be cognitive uncertainty [31]. Typically, this type of uncertainty is investigated using fuzzy logic [32,33]. Therefore, fuzzy classifiers can be considered an acceptable solution for classification problems in precision medicine. Taking into consideration the interpretability of the result, this should be a fuzzy classifier based on a decision tree. At the same time, a new method for classifier induction should allow its development for the different types of initial data. In particular, we intend to consider and systemize classification signals [29,34], large-dimensional data [35,36], numeric data [37,38] and linguistic data [28,39]. An analysis of these studies shows that there are differences in the pre-processing steps of the initial data. A fuzzy classifier can be inducted, based on one method for the different types of initial data, if these data are transformed into fuzzy data at the pre-processing step.

In this paper, a new method for the classification of heterogeneous, incompletely specified, and uncertain data is proposed. The incompleteness and uncertainty of data are taken into account by the use of a fuzzy classifier because a data process based on fuzzy logic enables the achievement of the best result in the case of cognitive uncertainty. The heterogeneous initial data in the proposed method are processed by the special preliminary transformation of the initial data of different types into fuzzy data. This transformation is similar to data pre-processing for signal classification, based on a fuzzy classifier [29]. In the case of signal classification, the data pre-processing includes the feature extraction of the signal [40], feature selection [41], fuzzification [42,43], and classification. The large dimensional data can be transformed by feature selection and fuzzification methods before they are classified. Numeric or linguistic data need fuzzification via the appropriate methods [44,45]. Different fuzzy classifiers can be used in this method. However, in this paper, we consider one of the possible classifiers, namely, the fuzzy decision tree (FDT). This choice is motivated by the good interpretability and visibility of the results from FDT. Another advantage of this classifier is the possibility of using it to process data of both small and large dimensions. In this study, the various FDT applications for some medical problems are summarized. An analysis of the efficiency of classification based on fuzzy classifiers shows that these classifiers are effective and yield the best accuracy in terms of classification, in comparison with crisp classifiers. The proposed method also allows FDT induction regarding different types of data. The novelty of this method is the universalization of the classification, which improves the compatibility of the mutual analysis of classification results from different resources.

This paper is organized as follows. The specific medical data of selected types for use in the proposed method are discussed in Section 2. The structure and principal steps, along with the procedures for every step of the proposed method, are presented in Section 3. A detailed analysis of every introduced procedure is presented in Section 4. In Section 5, examples of the application of the proposed method with different data type classifications are discussed. In this section, the classifications of EEG signals (signals), laboratory data (large dimensional data), clinical data (numeric and categorical data), and expert data (fuzzy data) are presented. Finally, an analysis of the classification metrics for every problem is introduced to show the efficiency of the proposed method.

## 2. Data Processing Specifics

### 2.1. Medical Data

The data used in precision medicine are big data. The typical characteristics of big data are volume, variety, and velocity (the “three Vs” of big data), which are then supplemented with such properties as value, veracity, and variability (the “six Vs” of big data) [46,47,48]. In big data processing, an emphasis is often placed on their large volume and the speed of occurrence (velocity). However, in the case of medical data, their heterogeneity is no less important and should be taken into account first [2,3,46]. The heterogeneity (variety) of medical data results from the use of a variety of available sources. Precision medicine uses comprehensive molecular diagnoses, which are based on the analysis of omics data (genotype, protein expression, RNA expression, etc.) [6]. Progress in IT and the emergence of the concept of Industry 4.0 have enabled the next step in medicine development, which is Healthcare 4.0. From the point of view of medical data analysis, the types of data involved in the processing are increasing [3], and the application of intelligence analysis methods is a distinguishing characteristic of Healthcare 4.0. The sources of data in this domain might be sensors, images, gene arrays, laboratory tests, free text, demographic data, etc. (Figure 2). This means that different types of data are involved in the work of processing and analyzing their significance. As a rule, different types of data require different methods for their processing: the processing of omics data is different from the processing and analysis of clinical data or signals from sensors, and special methods are used for each of these types. Of course, there are fundamental approaches that are used for processing most of the different types of data (such as classification based on a neural network), but there are significant differences in the pre-processing of each data type, and in the interpretation of the results. This can be illustrated by the review of precision medicine data processing presented by the authors of [6]. In this context, a problem with the comparability and compatibility of the obtained results is revealed [49,50]. Decisions regarding the comparability and compatibility problem can cause the additional transformation of the result and may increase the computational complexity of data processing [51] or uncertainty regarding the eventual decision [52]. 

However, uncertainty not only arises as a result of the processing of medical data but is also inherent in the initial medical data. There are many factors that can cause uncertainty [53]. It can result from the poor quality of data measurement or evaluation, where the values of the initial data should be considered according to likelihood. Uncertainty can be caused by the incomplete specification of the data, where collected data contain missing values because some situations (samples) cannot be measured or evaluated. One more cause of uncertainty can be from data pre-processing, where, for example, a dimensional reduction can result in the loss of some information [54,55].

According to a previous study [31], uncertainty can be interpreted as being both statistical and cognitive. This statistical uncertainty is related to the randomness of nature and can be mathematically represented in terms of probability theory. Cognitive uncertainty is related to the level of uncertainty associated with cognition or the abstraction of reality [56]. Cognitive uncertainty includes such types of uncertainty as ambiguity and vagueness. Vagueness is the uncertainty due to “borderline situations”, which is correlated with the difficulty of making exact decisions in borderline situations. Ambiguity is the uncertainty due to “alternatives” that exist in cases where the choice between alternatives is not exactly specified or some alternatives can be explained in a similar way. In many cases, the ambiguity and vagueness of medical data can be caused by experts’ knowledge, which is typical in healthcare [53,56]. The incompleteness of such data can be expensive or even dangerous for patients. One typical approach for taking into account the uncertainty of different types of data is the representation of information as fuzzy data [57,58].

Therefore, the development of medical applications should take into account heterogeneous and uncertain medical data. In this paper, we propose a new method for medical data classification that allows the processing of different types of data, based on a single platform that can take into account the uncertainty and incompleteness of medical data. The specific feature of the proposed method is the use of the fuzzy-based processing of the data, which takes into consideration the uncertainty of the initial data for the resulting analysis. 

### 2.2. Fuzzy Logic

Fuzzy logic introduces the notion of a membership degree [59]. A fuzzy set, A, with respect to a universe, U, is characterized by a *membership function*, *μ*_A_: U → [0, 1], assigning an A-membership degree, *μ*_A_(*u*), to each element of *u* in U. *μ*_A_(*u*) gives us an estimation of *u* that belongs to A. For *u* ∈ U, *μ*_A_(*u*) = 1, which means that *u* is definitely a member of A, while *μ*_A_(*u*) = 0 means that *u* is definitely not a member of A, and 0 < *μ*_A_(*u*) < 1 means that *u* is partially a member of A. If either *μ*_A_(*u*) = 0 or *μ*_A_(*u*) = 1 for all *u* ∈ U, A is a crisp set. In the opposite scenario, A is a fuzzy set. The cardinality measure of the fuzzy set A is defined by M(A) = Σ*_u_*_∈*U*_ *μ*_A_(*u*), which is a measure of the size of A.

The degree of membership allows specific elements to be partial members of a fuzzy set. It presents a contrast with traditional set theory because a classical set is defined by crisp (exact) boundaries. Therefore, there is no uncertainty about the location of the set boundaries. A fuzzy set is defined by its ambiguous boundaries. Hence, there exists uncertainty about the location of the fuzzy set boundaries. Partial membership is represented by a membership degree that can be between 0 and 1, including both 0 and 1. Fuzzy logic provides very valuable flexibility in terms of reasoning, which makes it possible to take into account the various inaccuracies and uncertainties.

Fuzzy logic enables the processing of this uncertainty but, at the same time, provides for the use of special methods and algorithms for data analysis. In addition, the initial data should be transformed into fuzzy data. These conditions result in the development of new methods for medical data analysis, based on the use of fuzzy logic. Below is one of the possible methods for medical data analysis based on the classification methodology.

## 3. A New Fuzzy-Based Method for the Classification of Medical Data

The methods used for medical data processing in precision medicine or in Medicine 4.0 should allow for the analysis of heterogeneous data (Figure 2). From the point of view of data analysis, the heterogeneity of the initial or source data is primarily associated with their processing. This process is most often defined as data pre-processing. Depending on the data type, the data can be transformed into another type, reduced, and cleared of noise and non-essential information. Depending on the procedures used in pre-processing, preliminary data can be interpreted as signals, images, high-dimensional data, numerical data, and expert data. Each type has its own pre-processing procedures. In this paper, the analysis of heterogeneous medical data according to classification is considered. A fuzzy classifier is used for the purposes of data analysis, to reduce the influence of pre-processing procedures on the classification result. The merging of different types of data is carried out by converting them to fuzzy data.

**Figure 3:**
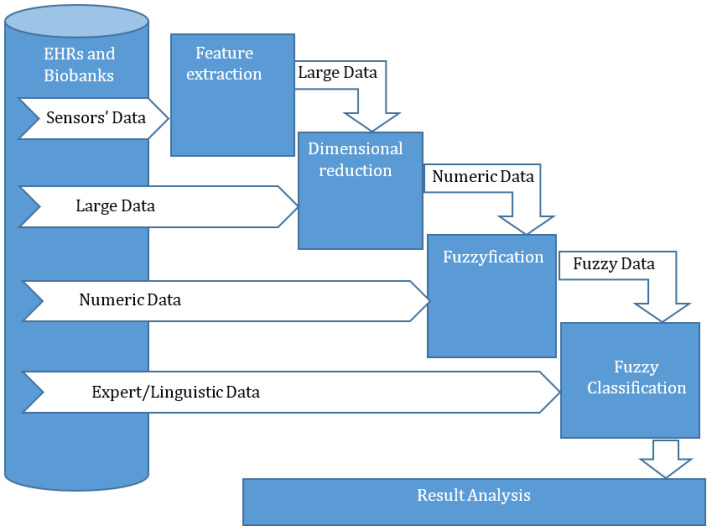
The structure of a new method for the classification of heterogeneous and uncertain medical data.

The core of the proposed method is a fuzzy classifier (Figure 3). As was shown in previous studies [29,30,32,33], the use of a fuzzy classifier makes it possible to increase the accuracy and efficiency of the analysis of incompletely specified and uncertain data [30,32] or signals [29]. In previous studies [54,55], the authors have shown that the pre-processing of a signal for its classification leads to the loss of some information. This loss is not large, but it can have an influence on the classification efficiency. The transformation of the pre-processing result into the fuzzy domain extends the classification attribute value borders and decreases the impact of lost information. In other studies [29,37] the efficiency of fuzzy classifiers for use with large dimensional data has been investigated.

The typical data pre-processing seen in signal classification includes the procedures of feature extraction and feature selection (dimension reduction). The procedure regarding feature extraction enables the discovery of information that can effectively reflect the specifics of the analyzed signal and identify its classification. The pre-processing of large dimensional data only needs to achieve dimension reduction, which can be performed via a procedure similar to signal classification. A dimension reduction procedure deletes unnecessary or redundant features by evaluating their usefulness and impact on classification efficiency. Feature selection and, as a result, the reduction in dimension of classified data, are required to simplify data visualization and comprehension and to minimize the time needed for classifier induction by decreasing the training time. The use of fuzzy classifiers determines that the input data for the classification should be fuzzy. Therefore, other types of input data (attributes) should be transformed into fuzzy data via fuzzification. The transformation enables the introduction of fuzzification to the proposed method. This procedure transforms each numeric attribute value into a fuzzy value with a membership function and is used for numeric data to achieve the unification of the utilized classifier. The expert (linguistic) data can be classified without pre-processing.

The structure diagram in Figure 3 shows that the proposed method enables the classification of data of various types, such as signals, high-dimensional numerical arrays, numerical data, and expert data. All these types of data are converted to fuzzy data using sequentially applied pre-processing procedures. The classifier is developed according to specified machine-learning algorithms for every type of data. The initial data for a classifier development create a table (dataset) that represents the fuzzy values of the output target attributes, depending on the fuzzy values of input and independent attributes. This table does not include all the possible combinations of values for the input attributes. Therefore, this table is not a decision table and cannot be used for the representation of complete knowledge about this object or process. The developed classifier permits the creation of a decision table for the investigated object or process, for analysis, or for use in the classification of a new sample. 

Each of the pre-processing procedures can be implemented, based on various algorithms and methods. It should be noted that the modification of these procedures can have a significant impact on the classification result. Unfortunately, the choice of the most efficient implementation method for each procedure is determined experimentally for each studied dataset [60,61,62]. There follows a brief overview of the most commonly used procedures of pre-processing.

## 4. Principal Steps of the New Method

### 4.1. Feature Extraction

Feature extraction is the first procedure in the pre-processing of a sensor’s dataset or signal [40]. The specific features of the signal should be extracted at the first step of signal pre-processing. The major goal of feature extraction is to obtain information that can effectively reflect the specifics of the analyzed signal and identify its class. Moreover, sensory data or signals are represented in the time spectrum, which is not suitable for classification without additional transformation. The accuracy of classification is mostly determined by the extracted features of the signal.

The feature extraction of sensor data or a signal is mostly implemented based on the methods in three groups: time, frequency, and time-frequency domains [63,64]. Some researchers consider a further group, namely, nonlinear feature analyses [65]. The methods of transformation of a signal in the time-frequency domain are the most commonly applied techniques for signal feature extraction.

Time-domain features are computed based on the signal amplitude according to time. Therefore, these methods are known as amplitude-based methods for feature extraction. The advantage of these methods is their low computational complexity. Moreover, this type of analysis often does not require additional signal transformation. There are many techniques used in the time domain for signal feature extraction. These methods include the histogram analysis method [66], higher-order crossing [67], and principal component analysis (PCA) [68]. Amplitude analysis can also be based on statistical features [69].

Features extraction based on Fourier transform methods [70] (fast Fourier transform and discrete Fourier transform) is conducted in the frequency domain (Table 1). Fourier-based and other frequency-based domain analysis methods convert time-domain signals to frequency-domain signals, in order to extract the frequency-domain features. These transforms are often used for EEG signal analysis and classification [70,71,72]. In addition to Fourier transform, the group of frequency-based feature extraction methods includes various techniques for the analysis of power spectral density (PSD) [71] and differential entropy (DE) [72].

The time- and frequency-based analysis methods used here are effective for linear and quasi-stationary signals [64]. In healthcare, signals are not linear and are quasi-stationary, for example, EEG signals. Time-frequency domain analysis methods (spectral methods) combine information from the time and frequency domains and permit time-frequency domain-localized analysis. The most effective and widely used methods of analysis in this group are wavelet transform [73] and Welch’s method [74]. Short-time Fourier transform (STFT) [75] and the Hilbert Huang transform (HHT) [76] are also essential time-frequency domain analysis methods.

There are studies in which new methods or combinations of previously known methods are developed for effective feature extraction of the signal. A new method for feature extraction for EEG signals, based on a combination of Fourier and Wavelet transforms using fuzzy entropy, is developed in an earlier study [77]. The authors of [78] propose a new time-frequency transformation by using joint approximate diagonalization for feature extraction. 

A very brief overview of the feature extraction of sensory data or signal methods is presented below. Here, it should be noted that the determination and development of the most effective feature extraction procedure is carried out experimentally for each type of signal. The feature extraction procedure is very important in terms of data pre-processing. However, the data obtained after this procedure have a large dimension, which must be reduced using the following procedure to ensure efficient classification.

### 4.2. Dimensionality Reduction

Dimensionality reduction or feature selection represents the next step in data pre-processing. This procedure can be used after the process of feature extraction or for processing initial data of larger dimensions. Feature selection is a procedure that is used to delete unnecessary (or redundant) features by evaluating their usefulness and their impact on classification efficiency in order to obtain the best results, based on the least possible amount of processed data [41]. Feature selection and, as a result, a reduction in the dimension of classified data are required to simplify data visualization and comprehension and to minimize the time needed for classifier induction by decreasing the training time. 

The most frequently applied procedures for feature selection are based on the techniques of linear discriminant analysis (LDA) [79], principal component analysis (PCA) [80], and independent component analysis (ICA) [81]. These techniques can be used to map a high-dimensional input vector into a low-dimensional vector. This transformation changes the physical context and nature of the initial features, which is not appropriate for some classification problems. However, these techniques are effective for signal classification [54]. PCA represents a more universal method for signal classification [79]. The use of PCA transforms the initial feature matrix with *n* features, *Y = (Y_1_, …, Y_s_)*, into a new matrix, *X* = (*X_1_, …, X_n_*), in which the features (columns) are known as “principal components” and *s* ≥ *n* [79]. In order to reduce the dimensions of the matrix, we must select some of the most important principal components. The importance of the principal components can be defined using component variance. The variance of the components indicates variability in the data.

There are techniques for feature selection that do not transform the initial feature vector [82,83]. Feature selection methods permit the exclusion of secondary features and choosing those that will have the maximum impact on the classification accuracy. These methods can be considered as three groups [82,83]: the filter, wrapper, and embedded methods. 

Filter-based methods use ranking to select the relevant features. The relevance is evaluated based on mutual information, the Pearson correlation coefficient, or other criteria [84]. The computational complexity of these methods is lower, and they can be used for any classifier induction. Each feature is evaluated separately using filter methods; therefore, some features can be removed according to an individual evaluation but they may become relevant when combined with others, or features that are considered individually to be relevant may result in unnecessary redundancies. Wrapper algorithms [85] execute feature selection in the context of (and in conjunction with) the classification algorithm. These methods can be interpreted as an optimization algorithm that employs the classification results as the target function. The computational complexity of wrapper methods is high. Finally, embedded methods perform attribute selection in the process of classifier induction and depend on the classifier and algorithm of this induction [82]. 

### 4.3. Fuzzification

Fuzzification is the procedure for data pre-processing used in the proposed method, which transforms linguistic data [42,44] or crisp numeric data [43,45] into fuzzy data (fuzzy attributes). Fuzzy attributes have values that are defined using the membership function (see Section 2.2). Every fuzzy attribute, *A_i_*, consists of the defined number *m_i_* (*m_i_* ≥ 2) in linguistic terms. The *j*-th linguistic term of *A_i_* is defined by the fuzzy set *A_i,j_* (*j* = 1, …, *m_i_*). Many methods are used for the fuzzification of both types of data. One of the first methods used was proposed by Zadeh [59], based on the use of non-fuzzy cardinality. Popular methods for the fuzzification of linguistic data were first developed by Yager [56,86]. A new definition for a linguistic quantifier was introduced elsewhere [87], while a two-step method for fuzzification and fuzzy quantification was introduced by the author of [44]. A review and analysis of many methods for the fuzzification of linguistic data are laid out in Ref. [42].

There are several fuzzification methods for crisp data. In this case, fuzzification converts crisp numeric attributes (principal components) that have been obtained after dimensionality reduction into fuzzy attributes *A_i_* (*i* = 1, …, *n*). The numeric attribute *X_i_* is defined by a vector of real scalar values (*x*_1_, *x*_2_, …, *x_k_*, …, *x_K_*), where *K* defines the number of samples. The definition of this fuzzy set, *A_i,j_*, with respect to *X_i_* is created by a membership function, $\mu_{A_{i,j}}(x)$: *X_i_* → {0, 1}, which provides a membership degree $\mu_{A_{i,j}}(x)$ for each *x* (*x* ∈ *X_i_*). The membership degree $\mu_{A_{i,j}}(x)$ determines how strongly the element, *x,* is a member of the fuzzy set, *A_i,j_*. Formally speaking, fuzzy set *A_i,j_* is defined as an ordered set of pairs, $A_{i,j}=\left\{\left(x,\mu_{A_{i,j}}(x)\right),x\in X_{i}\right\}$, where:

(a) $\mu_{A_{i,j}}\left(x\right)=0$, if, and only if, *x* is not the member of set *A_i,j_*;
(b) $0<\mu_{A_{i,j}}\left(x\right)<1$, if, and only if, *x* is not the full member of set *A_i,j_*;
(c) $\mu_{A_{i,j}}\left(x\right)=1$, if, and only if, *x* is the full member of set *A_i,j_*.

Fuzzification of crisp data can be implemented based on *g*-fuzzification [88], an evaluation of the histogram of variables [89], entropy analysis [90], and clustering [43,91,92]. Well-known clustering-based methods for fuzzification include the K-means algorithm and the fuzzy C-means method (FCM). 

### 4.4. Fuzzy Classification

There are many different fuzzy classifiers, for example, the fuzzy naïve Bayes classifier [93], fuzzy classification rules according to the algorithm reported in Ref. [94], neural networks [95], and decision tree-based classifiers [27,28,29,96]. The choice of one of these classifiers is implemented by an experimental evaluation of result efficiency (including accuracy, sensitivity, availability, and other metrics [62,97]). The properties of the classifier can also have an impact on the choice. For example, neural networks are effective for large dimensional data and are not suitable for data from small samples. However, Bayes classifier or tree-based methods are effective for such data. Note that the influence of the procedures of pre-processing is possible regarding classification efficiency. Therefore, the evaluation of a specified classifier’s efficiency should be implemented for different procedures of pre-processing. In this paper, we propose a technique using a decision tree-based classifier, specifically, FDT [29,30]. This classifier has very good interpretability, which is important for the medical domain. 

There are many different methods for FDT induction [96,98]. In this study, FDT induction is implemented based on cumulative information estimation (CIE), which was introduced by the authors of [99]. CIE evaluates the degree of influence of the input attribute on the resulting (output) attribute, in the context of information theory. In contrast to an informational assessment, which takes into consideration the average values of each input attribute, the CIE technique allows us to take into consideration the values of all observations and the measurements of each input attribute. The structure of FDT (the sequence and correlation of nodes) is determined, based on the CIM. Additionally, when building a tree, the parameters a and b are used. The values for the pruning parameters α and β affect the size and efficiency of the inducted FDT [98]. The decrease in parameter α and the increase in parameter β result in an FDT extension (due to increasing the numbers of levels and nodes) and a decrease in the classification accuracy because of the redundant information (noise) involved in FDT. An increase in parameter α and a decrease in parameter β lead to a reduced FDT size and a decrease in classification accuracy because essential information can be lost. The calculation of CIM and the process of choosing pruning parameters α and β is discussed in Ref. [98] in detail.

A decision tree is a flowchart-like structure in which each internal node represents a check on an input attribute, each branch represents the outcome of this check, and each leaf node represents a value of the output attribute (class label). 

The fuzzy classification efficiencies are evaluated based on several metrics, which are computed according to the results of the following classifications (classification instance) [62,97]: true positive (TP), true negative (TN), false positive (FP), or false negative (FN). The metrics of this classification are calculated, based on the classification instance:

- Accuracy is the ratio of correctly predicted instances to all predicted ones:
  (1) $$Acc=\frac{TP+TN}{TP+TN+FP+FN}$$
- Specificity is the proportion of correctly identified of true negative results:
  (2) $$Spec=\frac{TN}{TN+FP}$$
- Sensitivity is the probability of a correct initial prognosis:
  (3) $$Sens=\frac{\mathrm{TP}}{\mathrm{TP}+\mathrm{FN}}$$
- Precision is the proportion of correct positive predicted instances to the total number of instances in terms of positive predicted instances:
  (4) $$Prec=\frac{TP}{TP+FP}$$
- The F1 score is the harmonic mean of sensitivity and precision:
  (5) $$Fsc=\frac{2TP}{2\mathrm{TP}+\mathrm{FP}+\mathrm{FN}}$$

## 5. Examples of Method Application

In this section, illustrative examples of the proposed method are introduced. Signal classification, based on the proposed method, is shown in the context of EEG signal classification to detect epilepsy seizures [29]. Laboratory research into tumors, based on an analysis of blood parameter classification, is considered an example of large dimensional data classification [35]. Numeric data classification is illustrated by the timing of tracheostomies in COVID-19 patients [37]. Expert data classification is considered for an analysis of the influence of the human factor in a medical team [30]. In this paper, we do not induct the FDTs for the classification of the EEG signal, the timing of tracheostomy in COVID-19 patients, and human factor influence in a medical team. Instead, we show how these FDTs were inducted in previous studies [29,30,37] to illustrate the application of the proposed method (Figure 3).

The initial data for the considered classifiers’ induction have been introduced in our previous studies on signal classification [29], large dimensional data [35], numeric data [37], and expert data [30]. In these studies, we have discussed the induction of FDTs in detail. In each of the studies on FDT induction, the calculation of CIM and the pruning parameters α and β are discussed in detail. The comparison of inducted FDTs with other classifiers is also considered. In this section, the results of previous investigations and comparisons are used to illustrate the proposed new method. 

The inducted classifiers used in this study are evaluated using Equations (1)–(5) for the creation of a machine learning model on future (unseen/out-of-sample) data. This metric calculation is based on the application of the hold-out method. The initial dataset is divided into 2 samples; then, we build a model with the first (training) sample. The second (testing) sample is used for verification of the built model. Usually, the proportion of the dataset division is 70:30. According to the second method in the evaluation of the classifiers’ efficiency, random subsampling, the previous hold-out method is applied many times to achieve different divisions of the initial dataset.

### 5.1. Signal Classification

The EEG signal has many applications in medicine for diagnosing disease [63,65,67,68,71,72]. Often, these signals are used for neurology diagnostics in epilepsy patients [29,68,78]. There are some public datasets to study these signal classifications, which include records (samples) of EEG signals. In a recent study [29], the dataset from [100] has been used for the FDT induction and classification of epileptic seizures. This dataset includes 500 samples, comprising 23.6-second signal records. The dataset consists of 5 subsets of 100 samples. These subsets are collected for different conditions and patient states and are indicated by the letters A, B, C, D, and E. Subsets A and B were collected to include those persons who do not suffer from epilepsy, in different conditions. These conditions comprise having the eyes open or closed because this affects the electrical activity of the brain. The difference between these subsets is that for subset A, the patients had their eyes open, while for subset B, their eyes were closed. The subsets C, D, and E were collected from persons who suffered from epilepsy. The signals in subset E were collected for patients at the time of a seizure. The subsets C and D were collected during seizure-free intervals. The difference between subsets C and D is the zones of the EEG signal recording: samples of subsets D and C were recorded from within the epileptogenic zone and the hippocampal formation of the opposite hemisphere of the brain, accordingly. The problem of EEG signal classification can be considered as a classification into two classes, as ((A, B) and (C, D, E)) or ((A, B, C, D) and E)). In the same study [Rab2022], a second type of classification has been studied: (A, B, C, D) and E.

The proposed method for this case includes all the procedures of pre-processing (Figure 3). The procedures of fast Fourier transform (FFT) are used for feature extraction, those of PCA for dimensional reduction, and those of FCM for fuzzification. The initial data from EEG signal records in this study are cut and collected in a set of 4000 2.95-second records. This set of 4000 records of EEG signals are analyzed using FFT. The Fourier specter transforms to 8 attributes via PCA. The number of principal components has been obtained, based on an experimental investigation: the decrease in the number of principal components decreases the efficiency of the classification process, while an increase in the number of principal components increases the computational complexity of classification. This experiment is discussed in more detail in an earlier paper [29]. The procedure of FCM permits the representation of eight principal components by eight fuzzy attributes. 

In Table 1, an evaluation of the inducted FDT for EEG signal classification, in comparison with other classifiers, is shown. The comparison of fuzzy and non-fuzzy classifiers shows that the classification accuracy of the fuzzy classifiers yields the best performance in comparison with the similar non-fuzzy classifier. An analysis of the metrics for FDT shows that this classifier offers the best results in terms of accuracy, sensitivity, and F1 score, along with the second-best results in terms of precision and specificity. According to the same study [29], the inducted FDT is the best classifier for the identification of epilepsy seizures, based on the EEG signal.

The classification of the EEG signal is effectively implemented, based on fuzzy classifiers, which confirms the effective application of the proposed method for signal classification.

### 5.2. Large Dimensional Data Classification

Data are considered highly dimensional if the number of attributes is larger than the number of instances. High-dimensional datasets are common in the biological sciences. Fields of study such as genomics and the medical sciences will often use both tall and wide datasets that can be difficult to analyze or visualize using standard tools. An example of high-dimensional data in the biological sciences may include data collected from hospital patients, recording their symptoms, blood test results, behavior, and general health, resulting in datasets with large numbers of features.

Future experimental studies should be implemented for large dimensional data. Scientific laboratory research into the diagnosis of a tumor based on an analysis of blood parameters can be considered as an example. This laboratory study was performed on rats; data were created after monitoring Sprague Dawley rats [35]. The animals were adapted to standard vivarium conditions (temperature, humidity, a regular light and dark regimen). Data included 78 instances (rats). Monitoring was based on rat blood analysis. Blood from all the experimental animals was collected at a single point and then analyzed. After blood analysis, we obtained 186 input attribute values for each rat. These rats were then divided into two groups: the control and tumor groups. The goal of the prediction was to find out if the animal was suffering from a tumor.

The analysis of this data, according to the proposed new method, includes pre-processing and classification based on a fuzzy classifier, which, in this case, is FDT (Figure 3). Here, the pre-processing consists of the procedures of dimensional reduction and fuzzification, PCA, and FCM. The initial data are transformed into a new set of principal components, which consists of 5 input attributes.

We made a comparison of the classification results of the proposed method and other non-fuzzy classifiers for the same initial data, using the decision tree, naïve Bayes, neural network, k-nearest neighbor, and support vector machine (SVM) techniques. The experiments were implemented in MATLAB. For each classifier, we ran a procedure to find the best combination of input parameters. The procedure was based on multiple runs of the classifier using different values for the input parameters. The best classification result identified is shown in Table 2.

The classification of the data from the laboratory research shows the best result, according to the considered metric for the fuzzy classifier, which is FDT. This study shows that the proposed method is effective for large dimensional data and the use of a fuzzy classifier is effective.

### 5.3. Numeric Data Classification

In an earlier study [37], an analysis of the timing of tracheostomy in COVID-19 patients was based on the classification of initial data via FDT. The analysis, which was based on FDT, was developed in an investigation by the authors of [100], and included the table of supplementary materials (https://www.ncbi.nlm.nih.gov/pmc/articles/PMC7673767/, accessed on 17 November 2020). The data were collected from 177 health records from anonymous COVID-19 patients in the ICU at Guy’s and St Thomas’ National Health Service (NHS) Foundation Trust. The initial data of the investigated dataset comprise 29 input attributes for two groups. The attributes of the first group were obtained as the baseline characteristics, while the attributes of the second group were obtained during 14 days of hospitalization. The analysis was focused on an evaluation of tracheostomy timing and the prediction of a patient’s survival.

Baseline dataset characteristics can be interpreted as input attributes that are obtained immediately after the admission of a patient or by an analysis of the patient’s health records. The attributes of the first part, which can be obtained immediately, are age, gender, ethnicity, body mass index (BMI), and the acute physiology and chronic health evaluation II (APACHE II) score. The baseline characteristics, drawn from the patient’s health records, may be the confirmation of diabetes mellitus, hypertension, ischemic heart disease, chronic obstructive pulmonary disease, asthma, and chronic kidney disease. One more input attribute of the baseline characteristics is thromboembolism (pulmonary, venous, or multiple). These attributes, which were identified based on the baseline characteristics, are categorical (except for age and BMI, which are numerical).

The second group of input attributes was collected from the patient’s vital signs, markers of acute respiratory failure, and serum-based biomarkers for the severity of the disease. These attributes were measured repeatedly at different time points (after 24 h of clinical care on days 7, 10, and 14). If a patient died or was disconnected from mechanical ventilation, the last measured values were used. These attributes are identified as PEEP (positive end-expiratory pressure), FiO2 (fraction of inspired oxygen), PaO2 (partial pressure of oxygen), the PF ratio, CRP (C-reactive protein), ferritin, D-dimer, temperature, vasopressors, RRT (renal replacement therapy), and ECMO (extracorporeal membrane oxygenation). The attributes of the second group are numerical.

The input attributes for the considered problem are both categorical and numerical. The categorical data regarding patients can contain ambiguity, for example, due to the problem of the unambiguous interpretability of information in health records. The measured numerical data can have vagueness, which, for example, can be caused by a measuring device error. Therefore, fuzzy classification is more acceptable for the considered problem of facilitating decision-making as to the optimal timing of tracheostomy for prolonged respiratory weaning in critically ill COVID-19 patients. 

The evaluation of different classifiers is shown in Table 3. In this study, for numerical data, the best decision is obtained with the fuzzy classifier (FDT). Therefore, we can see that the proposed method is also effective for numeric data analysis.

### 5.4. Expert Data Classification

The last example provided for the proposed method considered the problem of the development of a mathematical model for the human reliability analysis of a medical team. The analyzed team included a doctor and two nurses. The goal of this problem is to evaluate the impact of human (medical) error on patient safety [30] (see Table 8 in Section 7). The mathematical model for human reliability analysis should classify all possible performance levels of the team’s members into three classes: a fatal medical error (set at 0), some imperfection in the patient’s care (set at 1), and patient care without any complications (set at 2). Therefore, the output attribute B has three values: B_0_, B_1_, and B_2_. The number of input attributes is defined according to the number of team members, which is 3. Two attributes, A_1_ and A_2_, are interpreted here regarding the nursing performance and have only two possible states: error (state 0) and error absent (state 1). The doctor’s performance is considered attribute A_3_ and has four levels: from a fatal doctor error (set at 0) to the doctor’s perfect work (set at 3). The impact of the mistakes made by a doctor or nurse on patient safety must be indicated and evaluated, based on a method of human reliability evaluation.

The initial data comprise expert evaluations of all 10 samples [30]. These data are incompletely specified because the sum of all possible situations is 16. Every value for the performance of each team member is indicated by a certainty. These data can be considered fuzzy data; thus, pre-processing is not needed for fuzzy classifier induction in this case. Therefore, the FDT can be inducted based on the initial data, without the need for additional transformation. An evaluation and comparison with other classifiers are presented in Table 4. 

The definition of the initial data values with certainty permits their interpretation as fuzzy data. The fuzzy classifier is inducted without the pre-processing of initial data, but a crisp classifier induction needs the initial data de-fuzzification. This procedure causes the classification accuracy to decrease. Only fuzzy classifiers should be considered for the comparison. There are specifics to this data that comprise the small dimension of the sample and a small number of input attributes. For such data, the use of a neural network as a classifier is impossible. A neural network cannot be trained on such a small dataset. Therefore, one classifier was inducted for the comparison with FDT, which is a fuzzy naive Bayes classifier. A comparison of the metrics of the classification efficiency in Table 4 shows that the FDT is best suited for the considered problem. 

## 6. Discussion

In this paper, the specifics of the classification of various data are considered and a new classification method is proposed to classify certain types of medical data. In particular, the proposed method (Figure 3) permits the classification of signals (sensors data), large dimensional numerical data, numerical, linguistic, and categorical data, and fuzzy data. Depending on the data type, the number of successively used pre-processing procedures changes. Signal pre-processing includes feature extraction, feature selection or dimensionality reduction, and fuzzification. Accordingly, for large dimensional data, the procedures for feature selection or dimension reduction and fuzzification are applied. Pre-processing consists of only one fuzzification procedure for numerical, categorical, and linguistic data. The procedure of fuzzification is used in pre-processing for all data types. It is due to the presence of a fuzzy classifier in the proposed method. As shown in previous studies [29,30,37], fuzzy classifier exploitation can improve the efficiency of classification. It is caused by the loss of some useful information in the relevant step of data pre-processing; first of all, in the procedure of dimensional reduction or feature selection [54,55]. Transformation of the input attributes before the classification from crisp to fuzzy allows us to “smear” the crisp values and thereby expand their coverage range, leading to improved classification accuracy. It is necessary to note that the different procedures of pre-processing impact the classification result. Unfortunately, there is no way to predict in advance the effectiveness of a particular procedure for data pre-processing [60]. Therefore, for every one of the procedures used in pre-processing, a short review of possible methods for its implementation is introduced. In this study, the procedures of FFT, PCA, and FCM are used for feature extraction, dimensional reduction, and fuzzification, respectively.

The classification process for all the considered problems has been implemented, based on FDT. This classifier can be effective for processing data with numerous input attributes and data with some input attributes. It can also be used for data comprising very small samples (see Section 5.4) without additional transformation, and also offers good interpretability and visibility. Of course, other classifiers can also be used instead of FDT. Similar to pre-processing procedures, the performance and efficiency of any classifier for each particular case cannot be predicted [60]. Using FDT for the considered problems in this study yields acceptable efficiency.

In this paper, the classification problems of the EEG signal (Section 5.1), scientific laboratory research on the diagnosis of a tumor, based on an analysis of blood parameters (Section 5.2), the timing of tracheostomy in COVID-19 patients (Section 5.3), and the expert data analysis of possible medical errors in a team (Section 5.4) have been considered. The data in these problems are different: signals, large dimensional data, numeric and categorical data, and expert (fuzzy) data. The proposed method (Figure 3) has been used in all these problems. The comparison of classification Equations (1)–(5) for FDT and other classifiers (first, for crisp classifiers) shows the efficiency of the proposed method. 

For example, Table 1 (EEG signal classification), Table 2 (the diagnosis of a tumor based on an analysis of blood parameters), and Table 3 (timing of tracheostomy in COVID-19 patients) introduce the metrics of classification efficiency for several classifiers. The classification efficiencies are evaluated for the different data types: signal data (Table 1), numeric large dimensional data (Table 2), and numeric data of limited dimensionality (Table 3). The comparison of FDT and the decision tree inducted using C 4.5 methods show that these metrics are somewhat better suited for FDT for all data types. In one study [55], it has been shown that signal preprocessing before classification can cause a loss of useful information and, according to a more recent study [Rab2022], the transformation of preprocessing results in a fuzzy domain that permits the leveling out of this loss by expanding the classified value boundaries. Fuzzy classifiers have similar or better efficiencies regarding classification in comparison with crisp classifiers of all types: fuzzy naive Bayes classifiers for signal classification (Table 1) and numeric data (Table 3) have the best metrics for classification than crisp versions of the naive Bayes classifiers. A similar result is achieved in a comparison of the fuzzy multi-layer perceptron and the crisp multi-layer perceptron.

Among the considered examples, the one that depicts the analysis of medical errors using a human reliability analysis method should be noted. The initial expert data are indicated in the form of values with certainty, allowing us to interpret them as fuzzy data. Therefore, for this data, a fuzzy classifier should be used (Table 4). In this problem, the sample under study is very small (only 10 samples). Therefore, the choice of classifiers is very limited. In particular, neural networks for such a small sample cannot be used. FDT and the fuzzy naive Bayes technique have been inducted for this problem. The considered metrics indicate that the best efficiency was achieved by FDT (Table 4) and, in addition, it offers good interpretability of the result. This classifier was used in an earlier study [30] for the development of a mathematical model for a reliability analysis of the human factor in medical systems.

Therefore, the considered examples and the previous studies [29,30,37] show that our proposed method for the classification of different data types in smart and precision medicine, based on fuzzy classifiers, is efficient. 

## 7. Conclusions

The most frequently considered problem in medical applications and services is result prediction, which depends on new initial data based on decisions that are already known. This problem is typical for both AI-based medicine and precision medicine (Figure 1). In fact, this can be considered a classification problem. The specifics of modern medical applications and services demand that such a prediction or classification must be performed for different types of data, and, as a rule, the classification of different types of data is implemented using different methods and algorithms. Typically, differences in these methods are found in the pre-processing procedures regarding the initial data (input attributes).

In this paper, a new method for the classification of different types of data is proposed; in particular, the classification of signal, large dimensional, numeric, or expert (linguistic or fuzzy) data is shown to be possible. One of this method’s advantages is the use of well-known procedures for data pre-processing: feature extraction, feature selection or dimensional reduction, and fuzzification. This permits resources for the method development to be decreased. The novelty of this method is the fuzzy classifier technique used, along with the new interpretation and exploitation of known methods and algorithms to address the new problem of the classification of different data types based on a single classifier. The unification of the classifier will make it easier to combine and jointly analyze the results from data of various types. According to the experimental evaluations, fuzzy classifiers offer better classification efficiency for the considered data types. In this paper, FDT is applied as a fuzzy classifier because it offers good interpretability of the results in comparison with other classifiers. However, other fuzzy classifiers can also be used. In future investigations, the influence of different methods and algorithms regarding the procedures of data preprocessing will be investigated. The influence of a membership function on method efficiency will also be considered. 

In this paper, the proposed method was used to classify each data type separately. This suggests that additional procedures will be required to analyze data of different types that are mixed together. This is a disadvantage to the proposed method. One final restriction of this method is discrete data processing. Continuous data should be discretized before the classification proceeds, based on the proposed method. In further studies, the different data types under consideration will be combined into a single sample, not in order to consider the processing of each of the presented data types independently, but instead to provide for their joint processing. In further studies, the proposed method will be supplemented with other data types that are widely used in medicine. One of the first additional types of data to be considered will be medical images.

## Figures and Tables

**Table 1:** Comparison of the efficiency of the proposed method based on FDT and other classifiers for signal classification.

Classification Algorithm	Type	Accuracy	Specificity	Sensitivity	Precision	F1 Score
FDT	Fuzzy	0.995	0.993	0.996	0.981	0.989
Decision Tree, C4.5	Non-fuzzy	0.981	0.969	0.992	0.978	0.984
Fuzzy Naive Bayes classifier	Fuzzy	0.952	0.917	0.999	0.862	0.924
Naive Bayes	Non-fuzzy	0.962	0.928	0.987	0.949	0.968
Fuzzy Multi-Layer Perceptron	Fuzzy	0.948	0.889	0.997	0.919	0.956
Multi-Layer Perceptron	Non-fuzzy	0.942	0.881	0.991	0.913	0.950

**Table 2:** Comparison of the efficiency of the proposed method, based on FDT and other classifiers for large dimensional data.

Algorithm	Type	Accuracy	Specificity	Sensitivity	Precision	F1 Score
FDT	Fuzzy	0.872	0.892	0.854	0.897	0.875
Decision Tree, C 4.5	Non-fuzzy	0.859	0.892	0.829	0.895	0.861
Naïve Bayes	Non-fuzzy	0.833	0.757	0.902	0.804	0.851
Neural Network	Non-fuzzy	0.859	0.838	0.878	0.857	0.867
K-nearest Neighbor	Non-fuzzy	0.795	0.811	0.780	0.821	0.800
SVM	Non-fuzzy	0.846	0.784	0.902	0.822	0.860

**Table 3:** Comparison of the efficiency of the proposed method, based on FDT and other classifiers of numeric data.

Classification Algorithm	Type	Accuracy	Specificity	Sensitivity	Precision	F1 Score
FDT	Fuzzy	0.867	0.563	0.921	0.921	0.921
Decision Tree, C4.5	Non-fuzzy	0.835	0.357	0.91	0.9	0.905
Fuzzy Naive Bayes classifier	Fuzzy	0.816	0.143	0.921	0.872	0.896
Naive Bayes	Non-fuzzy	0.816	0.143	0.921	0.872	0.896
Fuzzy Multi-Layer Perceptron	Fuzzy	0.857	0.563	0.91	0.92	0.915
Multi-Layer Perceptron	Non-fuzzy	0.845	0.357	0.921	0.901	0.911

**Table 4:** Comparison of the efficiency of the proposed method, based on FDT and other classifiers, for expert data.

Classification Algorithm	Type	Accuracy	Specificity	Sensitivity	Precision	F1 Score
FDT	Fuzzy	0.934	0.456	0.823	0.893	0.944
Fuzzy Naive Bayes classifiers	Fuzzy	0.915	0.372	0.798	0.852	0.902

## Data Availability

The data for the experimental study in this paper are from previously published investigations [29,30,35,37] (see Section 5).
